# Imatinib use immediately before stem cell transplantation in children with Philadelphia chromosome-positive acute lymphoblastic leukemia: Results from Japanese Pediatric Leukemia/Lymphoma Study Group (JPLSG) Study Ph^+^ALL04

**DOI:** 10.1002/cam4.383

**Published:** 2015-01-31

**Authors:** Atsushi Manabe, Hirohide Kawasaki, Hiroyuki Shimada, Itaru Kato, Yuichi Kodama, Atsushi Sato, Kimikazu Matsumoto, Keisuke Kato, Hiromasa Yabe, Kazuko Kudo, Motohiro Kato, Tomohiro Saito, Akiko M Saito, Masahito Tsurusawa, Keizo Horibe

**Affiliations:** 1 Department of Pediatrics, St. Luke's International HospitalTokyo, Japan; 2 Department of Pediatrics, Kansai Medical University Hirakata HospitalOsaka, Japan; 3 Department of Pediatrics, Keio University School of MedicineTokyo, Japan; 4 Pediatrics, Graduate School of Medicine Kyoto UniversityKyoto, Japan; 5 Department of Pediatrics, Kagoshima University Graduate School of Medical and Dental SciencesKagoshima, Japan; 6 Department of Hematology and Oncology, Miyagi Children's HospitalMiyagi, Japan; 7 National Research Institute for Child Health and DevelopmentTokyo, Japan; 8 Department of Pediatrics, Ibaraki Children's HospitalMito, Japan; 9 Department of Pediatrics, Tokai UniversityIsehara, Japan; 10 Department of Hematology/Oncology, Shizuoka Children's HospitalShizuoka, Japan; 11 Department of Pediatrics, University of TokyoTokyo, Japan; 12 MPH Department of Health Policy, National Research Institute for Child Health and DevelopmentTokyo, Japan; 13 MPH Laboratory of Clinical, Epidemiological and Health services Research, Clinical Research Center, National Hospital Organization, Nagoya Medical CenterAichi, Japan; 14 Deprtment of Pediatrics, Aichi Medical UniversityNagakute, Japan; 15 Clinical Research Center, National Hospital Organization Nagoya Medical CenterNagoya, Japan

**Keywords:** Ph+ALL, children, imatinib, HSCT, MRD

## Abstract

Incorporation of imatinib into chemotherapeutic regimens has improved the prognosis of children with Philadelphia chromosome-positive acute lymphoblastic leukemia (Ph^+^ALL). We investigated a role of imatinib immediately before hematopoietic stem cell transplantation (HSCT). Children with Ph^+^ALL were enrolled on JPLSG Ph^+^ALL 04 Study within 1 week of initiation of treatment for ALL. Treatment regimen consisted of Induction phase, Consolidation phase, Reinduction phase, 2 weeks of imatinib monotherapy phase, and HSCT phase (Etoposide+CY+TBI conditioning). Minimal residual disease (MRD), the amount of BCR–ABL transcripts, was measured with the real-time PCR method. The study was registered in UMIN-CTR: UMIN ID C000000290. Forty-two patients were registered and 36 patients (86%) achieved complete remission (CR). Eight of 17 patients (47%) who had detectable MRD at the beginning of imatinib monotherapy phase showed disappearance or decrease in MRD after imatinib treatment. Consequently, 26 patients received HSCT in the first CR and all the patients had engraftment and no patients died because of complications of HSCT. The 4-year event-free survival rates and overall survival rates among all the 42 patients were 54.1 ± 7.8% and 78.1 ± 6.5%, respectively. Four of six patients who did achieve CR and three of six who relapsed before HSCT were salvaged with imatinib-containing chemotherapy and subsequently treated with HSCT. The survival rate was excellent in this study although all patients received HSCT. A longer use of imatinib concurrently with chemotherapy should eliminate HSCT in a subset of patients with a rapid clearance of the disease.

## Introduction

Progress in childhood leukemia treatment has raised the 5-year survival rate to as high as 80–90%, however, outcomes in Philadelphia chromosome-positive acute lymphoblastic leukemia (Ph^+^ALL) patients still remain poor 1,2. Arico et al. reported results of an international retrospective study comprising 610 Ph^+^ALL children treated with intensive chemotherapy without tyrosine-kinase inhibitors and observed 7-year event-free survival (EFS) and overall survival (OS) to be 32% was 45%, respectively 3. They also showed that allogeneic hematopoietic stem cell transplantation (HSCT) was beneficial. In another study which was the first large prospective cohort study of pediatric patients treated with chemotherapy and tyrosine-kinase inhibitor (TKI), the Children's Oncology Group (COG) assessed increased exposure to imatinib combined with chemotherapy in five cohorts 4. Forty-four children, who received continuous imatinib from consolidation to the end of treatment, had 3-year EFS of 80%. In this group, which excluded patients with induction failure, the outcome of children treated with HSCT was not better than those treated with chemotherapy plus imatinib. The excellent outcome of this cohort of patients was recently updated: 5-year EFS of 28 patients treated with chemotherapy alone was 70% 5. Results of an additional study were recently reported by the European intergroup study (EsPhALL) 6. They adopted a risk-stratified approach for treatment of patients on the basis of early response to therapy and found that the combination of imatinib and Berlin-Frankfurt-Munster (BFM) backbone intensive treatment was safe and possibly beneficial to patients, although 77% received HSCT.

While results of these previous reports showed overall improved outcomes associated with imatinib plus intensive chemotherapy in children and adolescents with Ph-positive ALL, a poor prognosis is still observed for some Ph^+^ALL patients. The variations in the response to therapy suggest that Ph^+^ALL is heterogeneous with regard to sensitivity to chemotherapy, TKI and HSCT 7. The amount of minimal residual disease (MRD) at HSCT was shown to be associated with the outcome of children with ALL after HSCT 8. In this context, serial analyses of MRD may aid in the selection of patients who could be treated with intensive chemotherapy protocols including a tyrosine-kinase inhibitor.

Here, we report results of the Japanese Pediatric Leukemia/Lymphoma Study Group (JPLSG) Ph^+^ALL04 study, which was conducted in the same era as the COG and EsPhALL studies. Our main objectives were to investigate the potential therapeutic role of using imatinib immediately before HSCT and to evaluate the utility of quantitative MRD assessments on EFS and OS.

## Patients and Methods

### Patients

Children diagnosed with untreated Ph^+^ALL between the age of 1 and 18 years were consecutively enrolled from November 2004 to May 2008 onto the JPLSG Ph^+^ALL04 study. Written informed consent was obtained from the parents or guardians and from the patients as appropriate for their age and conceptual ability.

Diagnosis of ALL was based on morphological, biochemical, and flow cytometric features of leukemic cells, including lymphoblast morphology on May- or Wright-Giemsa-stained bone marrow smears, negative staining for myeloperoxidase, and reactivity with monoclonal antibodies to B- or T-lineage-associated lymphoid differentiation antigens.

All patients with ALL were screened for diagnosis of Ph^+^ALL using RT-PCR. The presence of Ph-chromosome was further confirmed by standard karyotyping and/or FISH analysis for BCR–ABL fusion gene. Forty-four children with Ph^+^ALL were enrolled into the JPLSG Ph^+^ALL04 study within 1 week of initiation of treatment for ALL. However, two patients were not evaluable because Ph-chromosome was not detected either with standard karyotyping or FISH analysis; therefore, 42 patients were eligible for analysis. The median patient follow-up period was 5.2 years (range: 0.6–7.5 years).

### Treatment protocol

**Figure 1:**
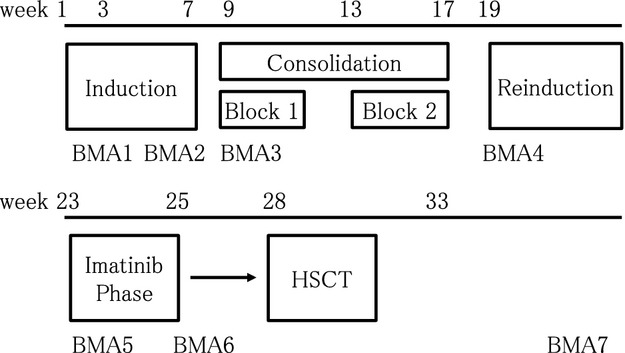
Ph^+^ALL04 protocol and timing of MRD detection. BMA1, day15; BMA2, day29; BMA3, before consolidation; BMA4, before reinduction; BMA5, before imatinib mesylate; BMA6, after imatinib mesylate; BMA7, 3 months after HSCT; BMA, bone marrow aspiration; HSCT, hematopoietic stem cell transplantation; MRD, minimal residual disease.

The protocol was approved by the institutional review boards of all participating institutions and by the Clinical Research Assessment Committee of the Japanese Society of Pediatric Hematology, which merged with the Japan Society of Pediatric Oncology and became the Japanese Society of Pediatric Hematology/Oncology in January 2012. Details of the treatment regimen of this single arm study are outlined in Figure1 and Table1. Chemotherapy regimen was based on the high-risk arm of TCCSG (Tokyo Children's Cancer Study Group) L99-15 9. Briefly, after five-drug induction therapy, consolidation therapy with high-dose cytarabine with asparaginase and BFM Ib-type was administered, followed by reinduction therapy with four-drug. After completion of reinduction therapy, imatinib monotherapy phase (2 weeks of imatinib at a dose of 340 mg/m^2^) was started, and all patients received allogeneic HSCT after imatinib phase. The conditioning regimen of HSCT was uniform across all patients and consisted of etoposide, cyclophosphamide, and total body irradiation 10,11. Prophylactic cranial irradiation was not employed. Imatinib was not used after HSCT. Remission was defined as the presence of fewer than 5% blasts with the recovery of hematopoiesis. Before and after each phase, MRD defined as the amount of BCR–ABL transcripts, was measured with the real-time PCR method (cut-off 50 copies/*μ*g RNA). Time points for MRD detection are shown in Figure1.

**Table 1:** Treatment scheme of Ph^+^ALL04.

Induction	Prednisolone 60 mg/m^2^ for 5 weeks, Vincristine 1.5 mg/m^2^ for five times, Daunorubicin 25 mg/m^2^ for four times, Cyclophosphamide 1200 mg/m^2^ for twice, l-asparaginase 6000 U/m^2^ for nine times, TIT for three times
Consolidation Block 1	High-dose cytarabine (2 g/m^2^ for eight times) with L-asparaginase 10,000 U/m^2^ once, TIT once, methylprednisolone 125 mg/m^2^ for eight times
Consolidation Block 2	Cyclophosphamide 1200 mg/m^2^ once, cytarabine 75 mg/m^2^ for 15 times, 6MP 60 mg/m^2^ for 21 days, TIT for three times
Reinduction	Dexamethasone 6 mg/m^2^ for 14 days, Vincristine 1.5 mg/m^2^ for four times, Doxorubicin 25 mg/m^2^ for four times, L-asparaginase 10,000 U/m^2^ for four times, TIT once
Imatinib monotherapy phase	Imatinib 340 mg/m^2^ for 14 days, TIT once
HSCT	TBI 12 Gy, Etoposide 60 mg/kg (BW <30 kg) or 1800 mg/m^2^ (BW ≥30 kg), Cyclophosphamide 60 mg/kg for twice

TIT, triple intrathecal therapy (MTX + Ara-C + hydrocortisone). Cranial irradiation was not given. HSCT, hematopoietic stem cell transplantation.

The study was registered in UMIN-CTR (Medical Information, University hospital Medical Information Network—Clinical Trials Registry, URL: http://www.umin.ac.jp/ctr/index-j.htm): UMIN ID C000000290.

### Statistical analysis

The primary endpoint of this study was to examine EFS and OS in the overall patient series and determine the efficacy of imatinib mesylate in children with Ph^+^ALL assessed by a molecular quantification technique. The sample size was determined by the Simon's two-stage minimax design 12. The lower limit of interest in the response probability was 20% and the desirable target level of response probability was 40%. The required sample size of eligible patients for the analysis was 33 for the alpha error at 0.05 and beta error at 0.20. The secondary objective was to evaluate the proportion of patients who received HSCT in the first complete remission (CR).

The duration of EFS was defined as the time from the initiation of therapy to either treatment failure (relapse, death, or diagnosis of secondary cancer) or the last day the patient was confirmed to be under remission. Patients who did not achieve CR after the first induction phase were considered to have failed at day 1. The probability of EFS and OS was estimated by the Kaplan–Meier method. All data analyses were performed using STATA® statistical software (version 11.0; StataCorp LP, College Station, TX). Follow-up data were actualized as of 31 May 2012.

### Role of the funding source

Novartis provided the study drug (imatinib mesylate). The sponsor had no role in study design, data collection, data analysis, data interpretation, or writing of the report. All authors had full access to all the data in the study and had final responsibility for decision to submit for publication.

## Results

### Patient characteristics and overall outcome of patients

**Figure 2:**
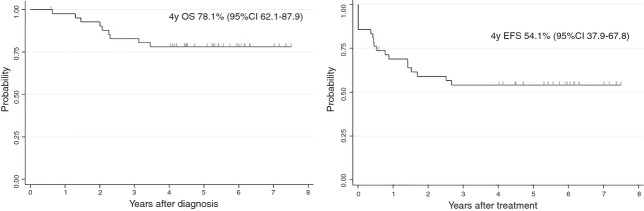
Overall survival of children with Ph^+^ALL (*n* = 42) (A) and event-free survival of children with Ph^+^ALL (*n* = 42) (B).

Of the 42 patients registered in the Ph^+^ALL04 study from 2004 to 2008 and included in this analysis, nine were girls and 33 were boys and the median age at diagnosis was 7 years (range 2–15 years) (Table2). Minor BCR–ABL fusion gene was detected in 33 children, whereas the major BCR–ABL fusion gene was detected in nine children. All patients had B-cell precursor ALL. Prednisolone response was assessed on day eight of steroid treatment. Thirty-three patients (79%) had less than 1000/*μ*L blasts in the peripheral blood and nine patients (21%) had equal or more than 1000/*μ*L blasts. Of the 42 patients, 36 (86%) achieved CR and 11 of these 36 patients also achieved MRD-negative after induction phase. A median follow-up period was 5.4 years. The 4-year OS (Fig.2A) and EFS (Fig.2B) rates among all patients were 78.1 ± 6.5% and 54.1 ± 7.8%, respectively.

**Table 2:** Characteristics of children with Ph^+^ALL (*n* = 42).

Median age at diagnosis (range)	7 years (2–15 years)
Girls/boys	9/33
White blood cell at diagnosis (range)	39 × 10^9^/L (1 − 681 × 10^9^/L)
CNS involvement at diagnosis yes^1^/no	3/39
Minor BCR–ABL/major BCR–ABL	33/9
Prednisolone response	
PGR^2^/PPR^3^	33/9
4-year EFS	54.1 ± 7.8%
4-year OS	78.1 ± 6.5%

1 CNS involvement was observed in three patients: all the three patients had blasts in the CSF.

2 PGR, prednisolone good responder (less than 1000/*μ*L blasts in the peripheral blood after 7 days of prednisolone treatment).

3 PPR, prednisolone poor responder (equal or more than 1000/*μ*L blasts in the peripheral blood after 7 days of prednisolone treatment).

### The efficacy of imatinib monotherapy

**Figure 3:**
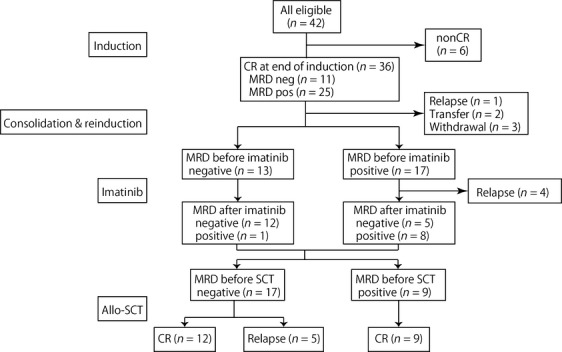
Flow diagram and MRD status of patients. MRD, minimal residual disease.

A flow diagram of the enrolled patients is shown as Figure3. Of 36 patients who achieved CR at the end of induction, the effects of the imatinib monotherapy phase was evaluable in 30 patients, as six patients excluded due to relapse (*n* = 1), transferring to non-JPLSG hospital (*n* = 2), and withdrawal (*n* = 3). There were 13 patients who had no MRD at the beginning of this phase, all of whom remained MRD-negative after imatinib monotherapy with the exception of one patient who had 100 copies/*μ*g RNA of BCR–ABL transcripts after 2 weeks of imatinib monotherapy. There were five patients who showed clearance of BCR–ABL transcripts after imatinib: the copy number of transcripts/*μ*g RNA of these patients was 450, 280, 250, 130, and 77, respectively. In the remaining 12 patients, three showed decrease of more than 1 − log transcripts: from 6600 to 140, from 39,000 to 1700, and from 1500 to 76, whereas four patients relapsed after this phase. Imatinib was well tolerated in all the patients.

### HSCT in the first CR

After 2-weeks of imatinib monotherapy, 26 patients underwent HSCT in the first CR, including 17 patients who were MRD-negative at the time of HSCT (Fig.2). The grafts were bone marrow from related donors for 10 patients, unrelated bone marrow for 10 patients, related cord blood for 1 patient, and unrelated cord blood for five patients. All the patients achieved engraftment, and no patients died because of complications of HSCT.

Five patients relapsed, all of whom were MRD negative before HSCT, Only two of the patients are alive after the second HSCT whereas the other three patients died because of treatment-related mortality. Twenty-one patients continued to be in 1st CR and MRD-negative for a median of 5.2 years after diagnosis. Of note, all the five patients who were treated with unrelated cord blood transplantation continued to be in 1st CR.

### Outcome of patients who did not have CR or who relapsed before HSCT

Of six patients who suffered induction failure, five patients were switched to an imatinib-containing chemotherapy treatment. Four of the five patients achieved CR, and all of these four patients received cord blood transplantation and remain in continued CR at 36, 42, 44, and 61 months after HSCT. Five patients relapsed before HSCT: one after consolidation and four after imatinib therapy. Three of the five patients achieved CR after imatinib-containing chemotherapy, and two of them are alive at 49 and 54 months after HSCT.

## Discussion

Addition of new drugs to conventional chemotherapy regimens is a challenging issue in treatment of leukemia. During the planning phase of the study, imatinib was not being used in children with Ph^+^ALL. The amount of MRD at HSCT was reported in relation to outcomes of children with ALL after HSCT 8. Therefore, we tested the hypothesis that the use of imatinib immediately before HSCT might be beneficial for children with Ph^+^ALL since it may reduce the amount of MRD at HSCT. We also measured BCR–ABL transcripts as a biomarker for imatinib response, to clarify its effect even when the disease was in CR. In this study, the chemotherapy regimen we employed was based on the previous high-risk treatment protocol of the TCCSG L-99-15 Study 9. The treatment strategy was effective in inducing MRD-negative status in 13 patients at the time of the imatinib phase and 26 of 42 patients (62%) achieved first CR at the time of HSCT (around 25–28 weeks after diagnosis). In consequence, it was not possible to perform a robust assessment of the efficacy of imatinib because the number of patients with detectable MRD (*n* = 17) at the beginning of the imatinib phase was much smaller than expected. Nevertheless, the imatinib therapy appeared to have antileukemic effects indicated by the observation that 47% of patients with detectable MRD at the beginning of this phase transitioned to MRD negative status after the short course imatinib treatment.

Twenty-six patients received HSCT in the first CR. Among them, MRD was negative at HSCT in 17 patient and all the five patients who relapsed after HSCT were MRD-negative at HSCT. In contrast, relapse was not observed in nine patients with a detectable level of MRD at HSCT. This suggests that the detection of MRD at HSCT was not related to the occurrence of relapse after HSCT in children with Ph^+^ALL. In adults with Ph^+^ALL, Lee et al. also described that the level of MRD at HSCT had little association with relapse after HSCT 13. However, in the Japan Adult Leukemia Study Group (JALSG) Ph+ALL202 protocol, the relapse rate was significantly lower among patients who were MRD negative at HSCT 14. More sensitive techniques, such as a deep-sequencing approach, may help to elucidate the significance of MRD at HSCT in patients with Ph^+^ALL 15.

The amount of MRD at the early phase of treatment for children with ALL distinguishes patients with good prognoses from those with poor prognoses 16,17. In our study, the amounts of BCR–ABL transcripts were prospectively monitored. In our cohort, among 26 patients who received HSCT at the first CR, 11 had MRD-negativity at the end of induction therapy, and two of the 11 patients relapsed after HSCT, while three of 15 patients with MRD-positivity at the end of induction suffered relapse after HSCT. Although the number of patients is small, the high/low status of MRD at the end of induction therapy did not seem to be correlated with relapse after HSCT. However, these data should be interpreted with caution because the method to detect MRD in our study and in JALSG was PCR detection of BCR–ABL transcripts, not an immunoglobulin/T-cell receptor (Ig/TCR) DNA-based technique or flow cytometry. In fact, Jeha, et al. recently reported that MRD detected with flow cytometry at the end of induction was dramatically reduced when TKI was incorporated into induction regimens 18.

In contrast, five of six patients who relapsed before HSCT had a high level of MRD of more than 10,000 copies/*μ*g RNA of BCR–ABL at least 1 month before hematological relapse. Zaliova, et al. also reported BCR–ABL transcript-based MRD enabled better and earlier prediction of relapse compared to DNA-based MRD 19. Taken together, the value of BCR–ABL-transcript-based-MRD has not yet been fully defined. Prospective studies in Ph^+^ALL patients comparing several methods of MRD assessment including BCR–ABL transcript, Ig/TCR-DNA, and flow cytometry is warranted. Although response to treatment based on MRD is considered essential for risk group stratification in current protocols for childhood ALL, the innate characteristics of leukemic cells, including additional karyotypic abnormalities^5^ and deletion of IKZF-1 20, might also be informative for the prediction of outcomes in patients with Ph^+^ALL.

Although OS was excellent in this study, an 86% induction rate appears unsatisfactory, in addition to six out of 36 patients in CR after induction phase experiencing a relapse before HSCT. The use of imatinib in the earlier phase of treatment, even in the induction phase, may be beneficial in children with Ph^+^ALL. Indeed, imatinib has been used in the induction phase of adult trials and has demonstrated an increase in CR rate 21. Furthermore, imatinib was successfully used in children from day 15 of induction in a recent SHOP study from Spain, but results were based on a small number of patients (*n* = 16) 22. In our study, all the nine patients treated with imatinib-containing chemotherapy as a salvage therapy achieved CR. Hyper-CVAD with imatinib was employed in seven of these nine patients. Hyper-CVAD with imatinib, which is widely used for adults with Ph^+^ALL 23, may be an alternative option for children with Ph^+^ALL as a salvage therapy. Detailed clinical course of these patients will be reported separately.

Both the COG and EsPhALL studies, which were contemporary to our study, showed that the use of imatinib concurrently with standard chemotherapy for ALL was safe and tolerable. Conceivably, HSCT may be omitted in a subset of patients who achieve deep remission status if earlier and longer use of imatinib is applied. In our study, all nine patients who were in first CR with a detectable level of MRD at HSCT continue to be in the first CR with negative MRD after HSCT. Based on our data, HSCT itself was safe and effective for children with Ph^+^ALL. Among 26 patients who were transplanted, no patients experienced treatment-related mortality in spite of the use of unrelated grafts in more than half of patients. It might be due to a uniform use of preconditioning regimen, a good selection of donors and an appropriate timing of HSCT. Eckert et al. also described the importance of standardization of HSCT procedure in the ALL REZ BFM 2002 trial 17. Since the late effects of HSCT are substantial, the indication of HSCT should be limited. However, HSCT is still an important modality for patients who are at high-risk for relapse, and conditioning regimen consisting of TBI, VP and CY may become a standard regimen for HSCT.

In conclusion, we interpret our results to suggest that the brief use of imatinib monotherapy on leukemic cells prior to HSCT may have a potential therapeutic effect which was demonstrated by 47% of MRD-positive patients transitioning to MRD negative status by the end of this phase. In addition, this was the first prospective trial to conduct HSCT in all children with Ph^+^ALL in first CR with a uniform conditioning treatment. Use of this protocol achieved an OS of approximately 80%. This result could serve as a basis for future trials aiming to reduce the rate of children who need be treated without HSCT. Finally prospective studies of Ph^+^ALL are warranted for the comparison of various MRD assessment methods, including BCR–ABL transcript, Ig/TCR-DNA and flow cytometry.

## Supporting Information

**Figure S1.** Kinetics of MRD during treatment. Each dot indicates each patient. Time points are shown in Figure 1. MRD, minimal residual disease.
