# Supplementary figures and images for: Imatinib use immediately before stem cell transplantation in children with Philadelphia chromosome-positive acute lymphoblastic leukemia: Results from Japanese Pediatric Leukemia/Lymphoma Study Group (JPLSG) Study Ph+ALL04
Source: Cancer Med. 2015 Jan 31;4(5):682–9. doi: 10.1002/cam4.383 (PMC4430261; doi:10.1002/cam4.383)

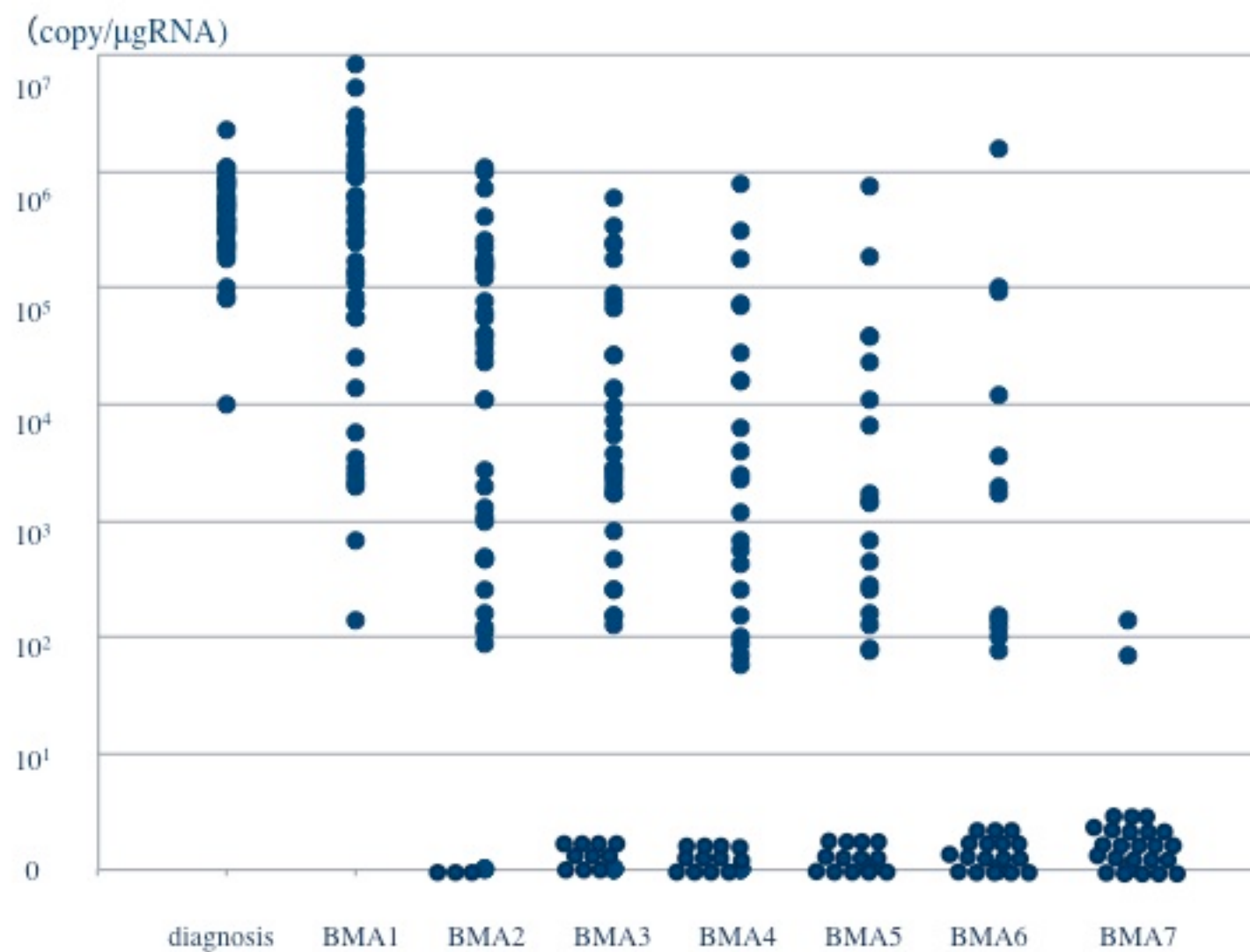

Figure 4

Supplement: Supplementary file 1 — Figure S1. Kinetics of MRD during treatment. Each dot indicates each patient. Time points are shown in Figure 1. MRD, minimal residual disease. [file cam40004-0682-sd1.pdf]
